# Capturing time-dependent activation of genes and stress-response pathways using transcriptomics in iPSC-derived renal proximal tubule cells

**DOI:** 10.1007/s10565-022-09783-5

**Published:** 2022-12-31

**Authors:** Paul Jennings, Giada Carta, Pranika Singh, Daniel da Costa Pereira, Anita Feher, Andras Dinnyes, Thomas E. Exner, Anja Wilmes

**Affiliations:** 1 Division of Molecular and Computational Toxicology, Chemistry and Pharmaceutical Sciences, AIMMS, Vrije Universiteit Amsterdam, Amsterdam, The Netherlands; 2 Edelweiss Connect GmbH, Technology Park Basel, Hochbergerstrasse 60C, 4057 Basel, Switzerland; 3 Division of Molecular and Systems Toxicology, Department of Pharmaceutical Sciences, University of Basel, Klingelbergstrasse 50, 4056 Basel, Switzerland; 4 BioTalentum Ltd, Aulich Lajos Street 26, Gödöllő, 2100 Hungary; 5 HCEMM-USZ Stem Cell Research Group, Hungarian Centre of Excellence for Molecular Medicine, Szeged, 6723 Hungary; 6 Seven Past Nine d.o.o., Hribljane 10, 1380 Cerknica, Slovenia

**Keywords:** Transcriptomics, Stress-response pathways, iPSC, Renal proximal tubular, Temporal gene expression, NAMs

## Abstract

Transcriptomic analysis is a powerful method in the utilization of New Approach Methods (NAMs) for identifying mechanisms of toxicity and application to hazard characterization. With this regard, mapping toxicological events to time of exposure would be helpful to characterize early events. Here, we investigated time-dependent changes in gene expression levels in iPSC-derived renal proximal tubular-like cells (PTL) treated with five diverse compounds using TempO-Seq transcriptomics with the aims to evaluate the application of PTL for toxicity prediction and to report on temporal effects for the activation of cellular stress response pathways. PTL were treated with either 50 μM amiodarone, 10 μM sodium arsenate, 5 nM rotenone, or 300 nM tunicamycin over a temporal time course between 1 and 24 h. The TGFβ-type I receptor kinase inhibitor GW788388 (1 μM) was used as a negative control. Pathway analysis revealed the induction of key stress-response pathways, including Nrf2 oxidative stress response, unfolding protein response, and metal stress response. Early response genes per pathway were identified much earlier than 24 h and included *HMOX1*, *ATF3*, *DDIT3*, and several *MT1* isotypes. GW788388 did not induce any genes within the stress response pathways above, but showed deregulation of genes involved in TGFβ inhibition, including downregulation of *CYP24A1* and *SERPINE1* and upregulation of *WT1*. This study highlights the application of iPSC-derived renal cells for prediction of cellular toxicity and sheds new light on the temporal and early effects of key genes that are involved in cellular stress response pathways.

**Supplementary Information:**

The online version contains supplementary material available at 10.1007/s10565-022-09783-5.

## Introduction

Toxicity evaluation of pharmaceutical compounds and environmental chemicals are to date still largely dependent on animal experiments. The goal to reduce and replace animal testing for toxicity studies is driven by multiple motivations, including scientific reasons (human relevance), the 7th amendment of the Cosmetics directive by the European parliament (EC No. 1223/2009), cost and time of performing these experiments, and ethical concerns. New Approach Methods (NAMs) (Escher et al. 2019; Parish et al. 2020; Fischer et al. 2020) are in vitro and/or in silico test methods that are being developed, utilized, and optimized under many settings, including large EU-wide consortia (e.g., EU-ToxRisk project (Moné et al. 2020), Riskhunt3r project (Pallocca et al. 2022) and the in3 consortium (https://estiv.org/in3/). NAMs are expected to better predict potential toxicity in humans and to accelerate the regulatory approval process. While there are still some limitations of NAMs, their potential role in safety assessment is promising and being recognized by regulators and scientists from academia and industry (Fischer et al. 2020; Rovida et al. 2021). In contrast to animal models for toxicity screening, which often rely on high doses of tested compounds leading to adverse effects that are then extrapolated to much lower doses that are expected for human exposure, in vitro toxicity studies are able to provide a more mechanistic approach (Bhattacharya et al. 2011). Transcriptomics and analysis of adaptive responses are becoming important tools in both understanding the mechanism of toxicity and determining the lowest observed effect concentrations (LOEC) of a test compound. Transcriptomics, whether based on mRNA microarrays or RNASeq (Bushel et al. 2018), is a highly sensitive endpoint, and changes in gene expression levels, including genes in cellular stress response pathways, are often observed at low concentrations before the occurrence of cell death. Cellular stress response pathways are well described and include among others pathways of oxidative stress, DNA damage, unfolded protein response, heavy metal stress response, hypoxia-induced stress, and inflammation (Jennings 2013; Jennings et al. 2013). Furthermore, several pathway analysis tools have been developed to cope with the analysis of such big datasets, including ConsensusPathDB (Kamburov et al. 2013; Kamburov and Herwig 2022), Ingenuity Pathway Analysis (Krämer et al. 2014), Reactome Pathway Knowledgebase (Jassal et al. 2020), and PathViso (Kutmon et al. 2015). Numerous studies report the usefulness of the application of (transcript)omics coupled to cellular stress response pathway analysis for the prediction of well-characterized toxic compounds in various human cell-based in vitro models, including renal proximal tubular cells (Wilmes et al. 2011, 2013, 2015; Jennings et al. 2012; Aschauer et al. 2015; Crean et al. 2015), hepatocytes (ter Braak et al. 2021; Ghosh et al. 2021; Grinberg et al. 2014; Limonciel et al. 2018), and neurons (Pallocca et al. 2016; Dreser et al. 2020; Delp et al. 2021). The use of sophisticated human cell systems is a unique selling point (USP) of modern in vitro applications, especially when the cells do not display cancerous geno- and pheno-types. Nevertheless, human cell lines represent only a single genetic donor, and human primary cells are often limited in availability and donor information. Hence, human-induced pluripotent stem cell (iPSC)-derived toxicity models may hold an even greater potential than other human cell systems, as they are widely available from numerous healthy donors and patients carrying genetic diseases. Therefore, they may also be useful for detecting individual and disease-specific responses after drug exposure. Several studies have demonstrated this, including studies in iPSC-derived cardiomyocytes and hepatocytes (Takayama et al. 2014; Blau et al. 2016; Grimm et al. 2018; Nunes et al. 2022). iPSC models also allow the possibility to test several tissues from the same donors.

Transcriptomic studies, despite being significantly cheaper in recent years, are still relatively expensive and thus often require a careful consideration in the number of samples that can be tested. This choice is often restricting the time points that are tested, with most studies choosing a single or a maximum of two or three time points per experiment, and a widely chosen initial time point, as observed in the literature, is 24 h of exposure. However, we would ideally like to know events that are proximal to the original molecular initiating event. Indeed, the temporal evolution of early events would be helpful in understanding mechanisms and could be used to develop (quantitative) adverse outcome pathways.

Here, we employed PTL cells and TempO-Seq transcriptomics (Limonciel et al. 2018) to study the temporal effects of five compounds, four that are known to activate common cellular stress response pathways and one that was chosen as a negative control (GW788388). PTL were exposed to test compounds for several time points between 1 and 24 h. Our aims were (1) to report on the temporal transcriptomics profiles of test compounds known to activate commonly reported stress response pathways and (2) to report on the applicability of the recently developed PTL cells in predicting toxicity using transcriptomics-based in vitro studies.

## Material and methods

### Cell culture and differentiation

iPSCs were maintained in mTeSR1 medium (StemCell Technologies 85850), with a daily medium replacement, on 6-well plates coated with Geltrex (ThermoFisher Scientific A1413302) in a humified incubator at 36.7 °C containing 5% CO_2_ and sub-cultured with EDTA (0.02% Versene, Lonza BE17-711E) twice per week. In this study, we employed the iPSC-line SBAD2 clone 1 that had previously been tagged with a GFP-reporter for HMOX1 expression (SBAD2-HMOX1-eGFP) (Snijders et al. 2021). This cell line was generated by BioTalentum, Gödöllő, Hungary. SBAD2 HMOX1-eGFP were differentiated into PTL cells on 96-well plates coated with Geltrex as previously described (Chandrasekaran et al. 2021). Briefly, cells were detached after 2–4-min incubation with accutase and centrifuged at 300 g for 5 min. After resuspension in differentiation medium (1:1 mixture of DMEM/F12 ThermoFisher Scientific 11966025 and ThermoFisher Scientific 21765029), 2 mM Glutamax and ITS (5 μg/mL, 5 μg/mL, 5 ng/mL, Sigma-Aldrich I1884) supplemented with 3 μM CHIR99021 (Abcam 120890), 1 μM TTNPB (Sigma-Aldrich T3757) and 10 μM rock inhibitor (Abcam ab120129) cells were seeded on Geltrex-coated 96-well plates at 35,000 cells per cm^2^. After 42 h, the medium was replaced with differentiation medium supplemented with 1 μM TTNPB. After an additional 30 h, the medium was replaced with proximal tubular medium (1:1 mixture of DMEM/F12, 2 mM Glutamax, ITS (5 μg/mL, 5 μg/mL, 5 ng/mL), 10 ng/mL EGF (Sigma-Aldrich E9644), and 36 ng/mL hydrocortisone (Sigma-Aldrich H0135) supplemented with 10 ng/mL FGF9 (ThermoFisher Scientific PHG0194). FGF9 was removed after 60 h, and cells were fed with proximal tubular medium. Cells were then fed every 2 to 3 days with proximal tubular medium until day 14.

### Immunofluorescence

For immunofluorescence experiments, PTL were differentiated in CellCarrier ultra-black 96-well plates. On day 14 of differentiation, PTL were fixed with 4% PFA (20 min), permeabilized with 0.1% Triton X-100 (10 min), and blocked with 5% BSA (1 h). Primary antibodies were applied for 1.5 h at RT in a DPBS 1% BSA solution and include megalin/LRP2 (1:100) (R&D systems MAB9578), ZO-3 (1:1600) (Cell Signaling Technology 3704), and occludin Alexa Fluor 594 (1:250) (ThermoFisher Scientific 331594). Secondary antibodies were applied (when applicable) together with and Hoechst 33342 (1:10.000) (ThermoFisher H3570) for 1 h at RT in a DPBS 1% BSA solution and include α-rabbit Alexa FluorTM 546 (1:1000) (ThermoFisher A10040) and α-mouse Alexa FluorTM 546 (1:1000) (ThermoFisher A10036). Cells were imaged using the Operetta CLS High-Content Imager (Perkin Elmer) with 40 × water objective, and images were exported using the Harmony software 4.8.

### Resazurin cell viability assay

Viability was measured as total cellular redox capacity using the resazurin reduction assay, conducted as previously described (Limonciel et al. 2011). Briefly, a 20 × stock solution was prepared by dissolving resazurin powder (Merck, R7017) in 0.1 N NaOH, bringing to the desired final volume with phosphate buffer and adjusting the pH to 7.8. After 24-h compound exposure, the medium was replaced with a 44 μM resazurin solution in cell culture medium and incubated for 2 h at 36.7 °C in a 5% CO_2_ humidified atmosphere. The fluorescent product of resazurin reduction, resorufin, was measured in a Clariostar plate reader at excitation/emission of 540/590 nm.

### Compound exposure, high-content imaging, and sample collection

On day 14 of differentiation, PTL were treated with either 0.1% DMSO (vehicle control) or with the following compounds: sodium arsenite (10 μM Sigma-Aldrich, S7400), amiodarone (50 μM Sigma-Aldrich, A8423), GW788388 (1 μM Sigma-Aldrich, SML0116, negative control), rotenone (5 nM Sigma-Aldrich, R8875), and tunicamycin (300 nM Sigma-Aldrich, 3516) over a temporal time course (1, 2, 4, 6, 8, 12, 16, 20, and 24 h). Three biological replicates were used. During compound exposure, cells were imaged for GFP expression (excitation at 488 nm and emission at 510 nm) in a High-Content Imager (HCI) Operetta (Perkin Elmer), and intensity of GFP signal was determined with the software Harmony 4.8 (Perkin Elmer). In details, the total GFP fluorescence intensity was given in relative units for each time point of the experiment. The measured fluorescence intensity was normalized by subtracting the fluorescence intensity from the first time point, where no GFP expression was detected. The normalized GFP fluorescence intensities were then plotted against time. In addition, cells were lysed in TempO-Seq buffer and collected for TempO-analysis as described before (Limonciel et al. 2018). Cadmium chloride treatment (5 μM Sigma-Aldrich, 202908) was used to induce GFP expression in the HCI experiment.

### TempO-Seq assay, data accessibility, and probe selection

Cell lysates were sent to BioClavis Technologies Ltd., Glasgow, Scotland, to perform TempO-Seq experiments using EU-ToxRisk v2.1 panel (3565 probes) (Mav et al. 2019; Limonciel et al. 2018). All samples were checked for and passed standard quality control. The raw data and meta data were accessed using the EdelweissData™ management system (SaferWorldbyDesign2021) using the Python script as described in Singh et al. (2021). The samples for both, treatment and corresponding controls, were selected for each of these compounds with additional filters, including cell type (iPSC-derived PTL cells) and time points (1, 2, 4, 6, 8, 12, 16, 20, and 24 h). This resulted in 27 samples for each compound including three biological replicates per time point and 27 samples for untreated controls, including three biological replicates per time point. The low read count probes were removed separately using the row sum threshold as 100 per probe across all samples per compound (including both treatment and control) before performing differential expression analysis (Love et al. 2014), resulting in 2789 probes for sodium arsenite, 2774 probes for amiodarone, 2794 probes for GW788388, 2787 probes for rotenone, and 2775 probes for tunicamycin out of the 3656 probes available in the TempO-Seq panel.

### Differential gene expression (DEG) analysis

The analysis was performed as previously described and is available in form of an R script (Singh et al. 2021). The data obtained after filtering the low read count probes was visualized, after R-log transformation using PCA and hierarchical clustering to see the variance between and among the different treatment and control normalized data and differential expression analysis groups (Love et al. 2014). The data was then normalized using the standard median-ratio method and on this was performed for treated group vs. corresponding control group for each compound (Love et al. 2014). The statistical values base mean, log2 fold change, adjusted *p* value (*p*-adj), *p* value, and log fold change error (lfcSE) were generated for each probe for each time point per compound. Additional filters were set to identify the most significantly expressed genes: *p*-adj was set to 0.01 and fold change >|2| (log2 fold change >|1|).

### Pathway enrichment analysis

Pathway analysis of the transcriptional response to test compound treatment was performed in the bioinformatic platform ConsensusPathDB V35 (CPDB) (Kamburov et al. 2013, 2022), for the time point inducing the highest number of significant DEGs. Enriched pathways were identified in CPDB through the over-representation analysis (ORA) of significantly expressed gene sets (fold change >|2|, *p*-adj < 0.01) compared to the predefined pathway-based sets included in the CPDB pathway database, which comprises several separated databases (Wikipathways, Smpdb, Kegg, Reactome, Pharmgkb, Pid, Biocarta, Ehmn, Humancyc, Inoh, Netpath, Signalink). ORA statistical analysis includes the calculation of a *p* value for each compound and pathway according to the hypergeometric test based on the number of matching genes between the input list (significant DEGs in pathway) and the reference list (total genes in pathway), taking in account the background list (tested genes in TempO-Seq panel). Cuts-off of significance for pathway activation include a minimum overlap of five entities between the input and reference list and a *p* value < 0.01. Significant *p* values were further corrected for multiple testing using the false discovery rate (FDR) method to generate *q* values used as a measure of ORA in this study. It is considered significant a *q* value < 0.05.

### BMDExpress analysis

The software BMDExpress2 has been developed as a tool to perform a dose–response modeling of transcriptomic data, to identify relationships between doses and changes in gene expression to support chemicals’ risk assessment studies (Phillips et al. 2019). In the present study, we have used the BMDExpress2 approach to estimate the time at which a specific biological change occurs upon treatment with a defined concentration of test compounds. Although the BMDExpress software was developed to predict benchmark doses (BMDs), the end results are parameterized curve fits of dose responses for single genes with associated statistics (Phillips et al. 2019). Fitting the curves to time responses does not change the mathematical model used. On this base, temporal transcriptional data collection up to 24 h was used to derive benchmark times (BMTs) using the same logic applied for the derivation of BMDs from a dose response transcriptional dataset.

For the generation of accumulation plots from a concentration range dataset, the deregulation of most genes follows a dose response trend, implicating that once a certain concentration has triggered the upregulation of a certain gene, a higher concentration will not shift the response back toward the baseline for the same gene, hence accumulating gene changes. For time responses, due to the characteristic of genes to be expressed in a specific time frame, not necessarily all genes follow a time-dependent deregulation, failing to fit the model. Thus, although accumulation plots of genes over time provide a valuable overview of the potency of the treatment applied, genes that demonstrate a differential deregulation within the time frame are not included; we therefore recommend analyzing the time responses of genes of interest in a case-by-case manner.

Temporal expression responses were prefiltered using a William’s trend test with a *p* value < 0.05 and fold change >|2| cut-off to select only significantly differentially changed genes. The analysis for the derivation of the BMTs was performed by fitting time response curves to ten parametric models used (Power, Hill, Exp2, Exp3, Exp4, Exp5, Linear, Poly2, Poly3, and Poly4), and best fitted model corresponding to the one giving the lowest Akaike information criterion (AIC) was selected. Best BMTs were further filtered for BMT < compound tested concentration, upper and lower bounds of confidence ratio (BMTU/BMTL) < 40, and best fitPValue > 0.1. Predicted fold changes associated with best BMTs were estimates in R (nplr package) using a weighted 5 − P logistic regression/progression model. Gene accumulation plots include only predictions inside the tested time course.

### Statistical analysis

Levels of statistical significance were calculated comparing treatment responses to control responses per time point using ordinary one-way ANOVA followed by a Dunnett post hoc test (*n* = 3). Significance codes indicate a *p*-adj: *** < 0.001, ** < 0.01, and * < 0.05 (Table 1). Data analysis was performed using RStudio R 4.2.1.

**Table 1:** Significance levels of genes per time point. Statistical analysis of significance for temporal responses of mRNA expression of selected relevant genes per pathway in Fig. 4. (a) Nrf2 oxidative stress response pathway, (b) unfolded protein response (UPR) pathway, and (c) metal stress response pathway. The data represents the mean of three experiments ± SD. Analysis was performed by ordinary one-way ANOVA with Dunnett post hoc test

a	Nrf2 pathway gene	Compound	1 h	2 h	4 h	6 h	8 h	12 h	16 h	20 h	24 h
	FTL	Amiodarone	0.771	0.961	0.005	0.006	0.023	0.033	0.167	< 0.001	< 0.001
		GW788388	0.690	0.856	0.850	0.566	0.991	0.014	0.823	0.210	0.182
		Rotenone	0.289	0.685	0.317	0.042	0.063	< 0.001	0.278	< 0.001	< 0.001
		Sodium arsenite	0.610	0.031	< 0.001	< 0.001	< 0.001	< 0.001	< 0.001	< 0.001	< 0.001
		Tunicamycin	0.870	0.967	0.720	0.899	0.804	0.348	0.955	0.003	0.004
	GCLM	Amiodarone	0.767	0.367	0.466	0.976	0.056	0.005	0.162	0.020	0.904
		GW788388	0.638	0.608	0.601	0.713	1.000	0.573	0.981	1.000	0.036
		Rotenone	0.072	0.069	0.259	0.861	0.508	0.034	0.765	0.224	0.919
		Sodium arsenite	0.256	< 0.001	< 0.001	< 0.001	< 0.001	< 0.001	< 0.001	< 0.001	< 0.001
		Tunicamycin	0.983	0.133	0.999	0.049	0.145	< 0.001	< 0.001	< 0.001	< 0.001
	HMOX1	Amiodarone	0.821	1.000	0.082	0.064	0.994	0.106	0.876	0.055	0.354
		GW788388	1.000	0.995	0.407	0.602	0.362	1.000	0.238	0.917	0.668
		Rotenone	0.908	1.000	0.979	0.065	0.924	0.284	< 0.001	< 0.001	< 0.001
		Sodium arsenite	< 0.001	0.000	< 0.001	< 0.001	< 0.001	< 0.001	< 0.001	< 0.001	< 0.001
		Tunicamycin	0.993	1.000	0.625	0.994	0.706	< 0.001	0.011	< 0.001	< 0.001
	MAFG	Amiodarone	0.726	0.998	0.948	0.162	0.007	0.042	0.053	0.021	< 0.001
		GW788388	0.765	0.076	0.226	0.679	0.335	0.259	0.371	0.987	1.000
		Rotenone	0.139	0.192	0.237	0.024	0.007	0.100	< 0.001	< 0.001	< 0.001
		Sodium arsenite	0.242	< 0.001	< 0.001	< 0.001	< 0.001	< 0.001	< 0.001	< 0.001	< 0.001
		Tunicamycin	0.891	0.306	0.910	0.249	0.035	0.055	0.052	0.059	0.200
	NQO1	Amiodarone	1.000	1.000	0.998	1.000	1.000	1.000	0.964	1.000	0.989
		GW788388	0.999	0.991	0.960	1.000	1.000	0.983	0.997	0.987	1.000
		Rotenone	0.961	0.993	0.888	0.987	0.823	0.901	0.926	0.909	0.992
		Sodium arsenite	0.998	0.997	0.235	0.122	0.029	0.012	0.004	0.005	0.004
		Tunicamycin	0.998	1.000	0.998	1.000	0.999	0.978	0.993	0.839	1.000
	SLC7A11	Amiodarone	0.980	0.028	0.965	0.114	0.796	0.396	0.019	0.504	0.463
		GW788388	0.183	0.574	1.000	1.000	1.000	1.000	1.000	0.996	0.984
		Rotenone	0.904	0.850	0.791	0.079	0.838	0.957	0.088	0.547	0.065
		Sodium arsenite	0.071	0.077	< 0.001	< 0.001	< 0.001	< 0.001	< 0.001	< 0.001	< 0.001
		Tunicamycin	0.496	0.647	0.521	0.002	0.046	< 0.001	< 0.001	< 0.001	< 0.001
	SQSTM1	Amiodarone	0.062	0.891	0.378	< 0.001	< 0.001	< 0.001	0.005	< 0.001	< 0.001
		GW788388	0.056	1.000	0.984	0.839	0.892	0.921	0.936	0.987	0.935
		Rotenone	0.084	0.999	0.955	0.064	0.998	0.718	0.037	0.008	< 0.001
		Sodium arsenite	0.171	0.039	< 0.001	< 0.001	< 0.001	< 0.001	< 0.001	< 0.001	< 0.001
		Tunicamycin	0.199	1.000	0.755	0.533	0.879	0.943	0.990	0.615	0.455
	TXNRD1	Amiodarone	0.653	1.000	0.994	0.752	0.180	0.330	1.000	0.878	1.000
		GW788388	0.999	0.447	1.000	1.000	0.491	0.520	0.999	1.000	0.313
		Rotenone	0.195	0.952	0.876	0.022	1.000	0.994	0.950	0.084	0.903
		Sodium arsenite	0.982	0.253	< 0.001	< 0.001	< 0.001	< 0.001	< 0.001	< 0.001	< 0.001
		Tunicamycin	0.325	0.430	0.799	0.792	1.000	0.283	1.000	0.577	0.992
b	UPR pathway gene	Compound	1 h	2 h	4 h	6 h	8 h	12 h	16 h	20 h	24 h
	ASNS	Amiodarone	0.339	0.573	1.000	0.003	< 0.001	0.000	< 0.001	< 0.001	< 0.001
		GW788388	1.000	0.249	0.843	0.591	1.000	1.000	0.998	0.129	1.000
		Rotenone	1.000	0.709	1.000	0.868	0.262	0.925	< 0.001	< 0.001	0.263
		Sodium arsenite	0.833	< 0.001	< 0.001	< 0.001	< 0.001	0.000	< 0.001	< 0.001	< 0.001
		Tunicamycin	0.838	0.020	0.003	< 0.001	< 0.001	0.000	< 0.001	< 0.001	< 0.001
	ATF4	Amiodarone	0.951	0.321	0.056	0.133	< 0.001	< 0.001	< 0.001	0.012	< 0.001
		GW788388	0.171	0.066	0.048	0.938	0.423	1.000	0.362	0.883	1.000
		Rotenone	0.471	1.000	0.085	1.000	0.168	0.121	0.003	0.004	0.007
		Sodium arsenite	0.340	< 0.001	< 0.001	< 0.001	< 0.001	< 0.001	< 0.001	< 0.001	< 0.001
		Tunicamycin	0.895	0.248	0.209	< 0.001	< 0.001	< 0.001	< 0.001	< 0.001	< 0.001
	DDIT3	Amiodarone	0.986	0.600	< 0.001	< 0.001	< 0.001	< 0.001	0.000	< 0.001	0.000
		GW788388	0.999	0.927	0.994	1.000	0.258	0.990	0.995	0.509	0.990
		Rotenone	1.000	0.381	0.106	0.004	0.011	0.194	0.000	< 0.001	0.000
		Sodium arsenite	0.008	< 0.001	< 0.001	< 0.001	< 0.001	< 0.001	0.000	< 0.001	0.000
		Tunicamycin	0.868	0.032	< 0.001	< 0.001	< 0.001	< 0.001	0.000	< 0.001	0.000
	DNAJB9	Amiodarone	0.999	1.000	0.500	0.002	0.611	0.745	0.535	0.039	0.087
		GW788388	0.997	0.991	0.751	0.406	1.000	0.896	0.971	0.982	0.891
		Rotenone	0.026	0.814	1.000	0.020	0.988	0.900	0.089	< 0.001	< 0.001
		Sodium arsenite	0.854	0.216	0.340	0.072	0.116	0.101	0.999	1.000	1.000
		Tunicamycin	0.813	0.998	0.266	< 0.001	< 0.001	< 0.001	< 0.001	< 0.001	< 0.001
	HSPA5	Amiodarone	1.000	0.128	0.881	0.079	0.005	0.003	< 0.001	< 0.001	< 0.001
		GW788388	0.965	0.021	0.132	0.780	0.170	0.692	1.000	0.297	0.936
		Rotenone	0.870	0.304	0.738	0.097	0.772	1.000	< 0.001	< 0.001	< 0.001
		Sodium arsenite	1.000	0.936	0.995	1.000	0.937	0.857	0.787	0.005	0.271
		Tunicamycin	1.000	0.119	0.016	< 0.001	< 0.001	< 0.001	< 0.001	< 0.001	< 0.001
	PPP1R15A	Amiodarone	1.000	0.992	0.378	0.015	< 0.001	0.033	0.005	< 0.001	< 0.001
		GW788388	0.656	0.098	0.076	0.324	0.995	0.822	0.894	0.062	0.774
		Rotenone	1.000	0.738	0.976	0.140	0.021	0.850	< 0.001	< 0.001	< 0.001
		Sodium arsenite	0.437	< 0.001	< 0.001	< 0.001	< 0.001	< 0.001	< 0.001	< 0.001	< 0.001
		Tunicamycin	0.999	0.808	0.998	0.011	< 0.001	0.075	0.002	< 0.001	0.019
	TRIB3	Amiodarone	0.718	0.988	0.999	0.000	0.000	< 0.001	< 0.001	< 0.001	< 0.001
		GW788388	0.782	0.598	0.944	1.000	1.000	1.000	1.000	0.282	0.894
		Rotenone	0.175	0.171	0.413	0.992	0.970	0.239	0.003	< 0.001	< 0.001
		Sodium arsenite	1.000	< 0.001	< 0.001	0.000	0.000	< 0.001	< 0.001	< 0.001	< 0.001
		Tunicamycin	0.943	0.831	< 0.001	0.000	0.000	< 0.001	< 0.001	< 0.001	< 0.001
	XBP1	Amiodarone	0.477	0.994	0.009	< 0.001	< 0.001	< 0.001	0.026	< 0.001	0.004
		GW788388	0.478	1.000	0.641	1.000	0.752	0.994	1.000	1.000	0.999
		Rotenone	< 0.001	0.901	0.275	0.247	0.993	0.697	0.051	< 0.001	< 0.001
		Sodium arsenite	0.008	< 0.001	< 0.001	< 0.001	< 0.001	< 0.001	0.029	< 0.001	0.007
		Tunicamycin	0.025	0.998	< 0.001	< 0.001	< 0.001	< 0.001	< 0.001	< 0.001	< 0.001
c	Metal pathway gene	Compound	1 h	2 h	4 h	6 h	8 h	12 h	16 h	20 h	24 h
	MT1E	Amiodarone	0.949	0.596	0.125	0.002	0.022	0.113	1.000	0.600	0.999
		GW788388	0.987	0.252	1.000	0.810	1.000	1.000	1.000	0.489	1.000
		Rotenone	0.008	0.161	0.277	< 0.001	0.051	0.836	< 0.001	< 0.001	0.551
		Sodium arsenite	1.000	< 0.001	< 0.001	< 0.001	< 0.001	< 0.001	< 0.001	< 0.001	< 0.001
		Tunicamycin	0.241	0.012	0.905	0.785	0.440	0.801	0.100	0.145	1.000
	MT1F	Amiodarone	0.999	0.999	0.182	0.127	0.032	0.071	0.559	0.248	0.236
		GW788388	0.207	0.905	0.989	0.996	1.000	0.996	1.000	0.883	0.888
		Rotenone	0.856	0.969	0.219	1.000	0.402	0.940	0.861	0.870	0.991
		Sodium arsenite	0.668	< 0.001	< 0.001	< 0.001	< 0.001	< 0.001	< 0.001	< 0.001	< 0.001
		Tunicamycin	0.623	0.948	1.000	1.000	0.170	1.000	0.511	0.998	0.410
	MT1G	Amiodarone	1.000	1.000	< 0.001	< 0.001	< 0.001	0.028	0.041	0.026	0.003
		GW788388	0.646	0.290	0.317	0.980	0.852	0.293	0.983	0.909	0.978
		Rotenone	0.123	0.040	0.003	0.017	0.031	0.102	0.055	0.016	0.111
		Sodium arsenite	1.000	< 0.001	< 0.001	< 0.001	< 0.001	< 0.001	< 0.001	< 0.001	< 0.001
		Tunicamycin	0.402	0.107	0.985	0.996	0.083	0.670	0.681	0.618	1.000
	MT1M	Amiodarone	1.000	0.506	< 0.001	< 0.001	< 0.001	0.099	0.008	0.630	0.004
		GW788388	0.822	0.804	0.999	0.383	1.000	0.995	0.988	0.914	0.473
		Rotenone	0.107	0.053	< 0.001	0.019	0.006	0.072	< 0.001	0.024	0.194
		Sodium arsenite	0.551	< 0.001	< 0.001	< 0.001	< 0.001	< 0.001	< 0.001	< 0.001	< 0.001
		Tunicamycin	0.694	0.350	1.000	0.961	0.147	0.420	0.522	0.263	0.999
	MT1X	Amiodarone	0.996	1.000	0.002	< 0.001	0.024	0.533	0.354	0.015	0.131
		GW788388	0.539	0.189	0.866	0.147	1.000	0.915	0.887	1.000	0.999
		Rotenone	0.361	1.000	0.995	0.938	0.032	0.228	1.000	0.088	0.007
		Sodium arsenite	0.790	< 0.001	< 0.001	< 0.001	< 0.001	< 0.001	< 0.001	< 0.001	< 0.001
		Tunicamycin	0.999	0.658	0.711	0.203	0.633	0.378	0.981	0.997	0.995
	MT2A	Amiodarone	0.996	1.000	< 0.001	< 0.001	< 0.001	0.029	0.115	0.002	< 0.001
		GW788388	1.000	0.952	0.263	0.929	0.988	0.683	0.997	0.681	0.708
		Rotenone	0.209	0.207	0.003	0.060	0.125	0.335	0.263	0.011	0.333
		Sodium arsenite	0.998	< 0.001	< 0.001	< 0.001	< 0.001	< 0.001	< 0.001	< 0.001	< 0.001
		Tunicamycin	0.384	0.358	0.845	0.741	0.101	0.851	0.364	0.769	0.825

## Results

### Differential gene expression analysis and pathway enrichment analysis

**Fig. 1:**
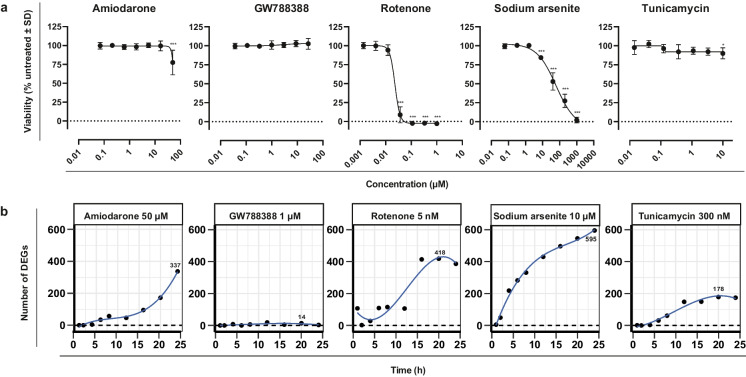
Effects of compounds on iPSC-derived proximal tubular cells (PTL). PTL were differentiated in 96-well plates for 14 days followed by compound exposure for up to 24 h. **a** Viability as measured by resazurin reduction in PTL treated with a dose-range of test compounds for 24 h. Data is expressed as percentage of 0.1% DMSO-treated control cells. The data represents the mean of 6 experiments ± SD. Statistical significance was analyzed by applying an ordinary one-way ANOVA followed by a Dunnett post hoc test. Significance codes indicate a *p*-adj: *** < 0.001, ** < 0.01, and * < 0.05. **b** Cells were lysed at different time points and mRNA changes were analyzed by TempO-Seq transcriptomics. Total number of deregulated expressed genes (DEGs) were calculated by comparing with 0.1% DMSO-treated control cells. Total number of significant DEGs (cut-off: fold change > |2|, *p*-adj < 0.01) are displayed per time point

**Fig. 2:**
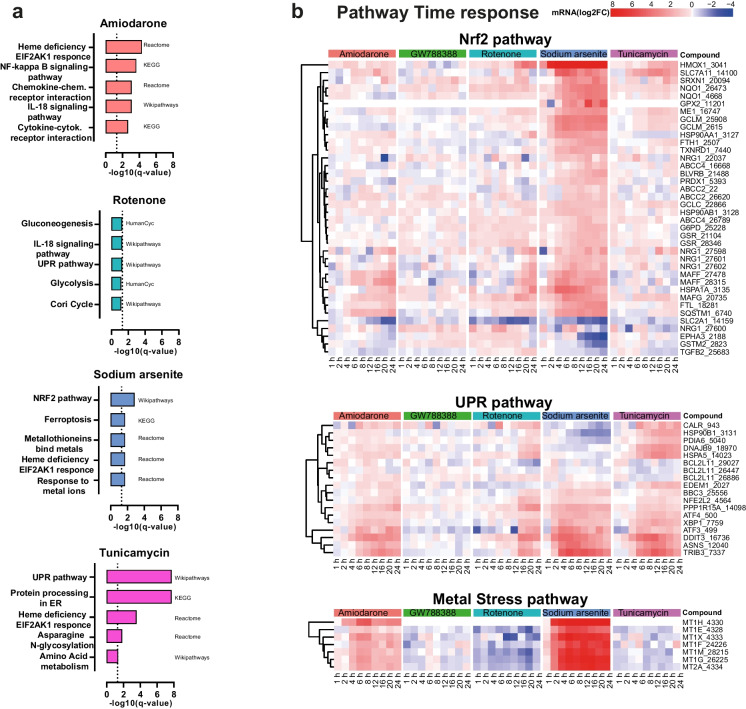
Pathway analysis in ConsensusPathDB. **a** Top five altered pathways upon treatment of PTL with 50 μM amiodarone, 10 μM sodium arsenite, 5 nM rotenone, and 300 nM tunicamycin at the highest DEG response time point. Pathway analysis of significant genes (fold change > |2|, *p*-adj < 0.01) was performed in the ConsensusPathBD bioinformatic platform by ORA analysis. Dotted vertical horizontal lines correspond to the threshold of significance for pathway alteration, *q* value < 0.05. **b** Heat map of genes associated with stress response pathways after treatment with compounds. Time responses of genes per pathway identified in the pathway analysis. Gene lists include all genes in the pathway significantly changed by at least one compound (fold change > |2|, *p*-adj < 0.01)

Kinetic profiles of key cellular stress response pathways that were upregulated in response to four well-characterized compounds in iPSC-derived PTL were analyzed using TempO-Seq transcriptomics. These include the anti-arrhythmia drug amiodarone, the metalloid sodium arsenite, the insecticide rotenone, and the antibiotic tunicamycin. As a negative control compound, the selective TGFβ type I receptor kinase (aka ALK5) inhibitor GW788388 was used. PTL were previously characterized for expression of proximal tubular markers and functional transport of P-glycoprotein (PGP) and megalin-facilitated endocytosis (Chandrasekaran et al. 2021) and a representative staining of megalin, occludin, and ZO3 in PTL derived from the SBAD2-HMOX1-eGFP cell line is shown in Supplementary Figure S1. Dose response curves of tested compounds were generated with the resazurin cell viability assay at 24 h after exposure. For TempO-Seq, all compounds were used at concentrations below IC10 values (Fig. 1a). TempO-Seq data was first analyzed using the filtered raw read counts of probes and PCA plots (Supplementary Figure S2) that showed good separation of individual groups for all compounds except for the negative control compound GW788388 that clustered with untreated control samples. Treated and untreated cells separated well in most cases, with early time points clustering closer to the control samples and later time points showing temporal progression of the changes. The four test compounds showed a significant increase of DEGs over time, with early responses as soon as 2–4 h and increasing numbers of DEGs by 24 h, whereas the negative control compound had a very mild effect with relatively few significantly DEGs (Fig. 1b). Amiodarone and sodium arsenite showed the highest number of DEGs (represented by 337 and 595 probes, respectively) at the 24 h time point. Tunicamycin and rotenone showed the highest DEGs (178 and 418 probes, respectively) at 20 h. GW788388 only showed a sum of 14 probes differentially expressed at any time point. Pathway enrichment analysis was performed using ConsensusPathDB to avoid bias from the pathway coverage of a specific pathway. To identify significantly overrepresented pathways, thresholds for the *p* value < 0.01 and *q* value < 0.05 were applied. The main over-represented pathways included metal stress response pathways (e.g., “metallothioneins bind metals” and “metal ion response”) in response to sodium arsenite (*q* value 1.98e − 02 for both pathways); Nrf2-mediated oxidative stress response pathways (e.g., “Nrf2 pathway”) in response to sodium arsenite (*q* value 1.43e − 03), and unfolded protein response (e.g., “UPR pathway”) and protein processing in ER (*q* value 1.95e − 08 for both pathways) in response to tunicamycin (Fig. 2a). Over-representation analysis in response to rotenone treatment included the UPR pathway, IL-18 signaling, and gluconeogenesis as the top pathways; however, non-significant *q* values (*q* values: 0.055) were obtained. Genes within these stress response pathways that significantly changed by at least one compound were selected and presented in the form of a heat map for all compounds (Fig. 2b). The fold-changes of DEGs (represented by color intensities in the heat map, with dark red showing the highest fold change increased genes and dark blue showing the highest fold changed decreased genes) appeared to be overall in line with the pathway *q* value and number of DEGs per pathway. Genes within the metal stress response pathway showed the highest upregulation in response to sodium arsenite (*MT1F* isotypes lfc 6.81 at 24 h). Interestingly, amiodarone also induced most genes within this pathway even though comparably lower fold changes were observed compared to sodium arsenite (*MTF1* isotypes lfc 1.81 at 24 h). The Nrf2-mediated oxidative stress response showed the highest fold changes in response to sodium arsenite, with *HMOX1* being the highest upregulated gene (lfc 9.98 at 24 h). Other compounds that did not meet *q* value settings for activation of the Nrf2 pathways due to lower number of DEGs, induced much lower lfc alterations, including for *HMOX1*. Nevertheless, tunicamycin and rotenone induced *HMOX1* at the late time points, but much lower lfc of other Nrf2 associated genes compared to sodium arsenite. The unfolded protein response pathway was significantly induced by three of the above compounds and showed the highest lfc in response to tunicamycin and sodium arsenite with *TRIB3* and *DDIT3* being the highest upregulated genes observed. Rotenone induced slightly lower fold changes, while the total number of DEGs was relatively high. Amiodarone induced a lower number of genes and therefore did not meet significant criteria for activation of the UPR pathway. Nevertheless, some genes, including *TRIB3* and *DDIT3* showed upregulation at several time points.

### HMOX1 reporter activation

**Fig. 3:**
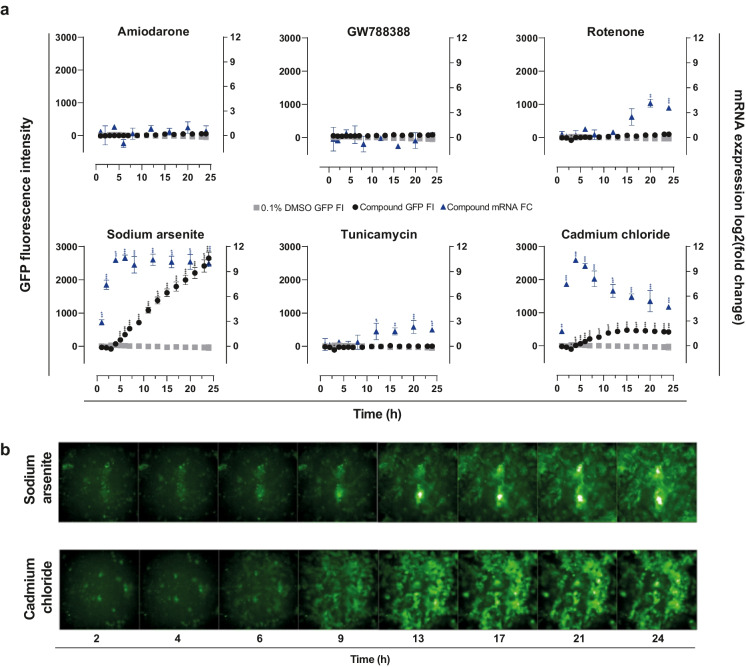
HMOX1 expression in response to treatment with compounds. PTL were differentiated in 96-well plate for 14 days and treated for up to 24 h with 50 μM amiodarone, 10 μM sodium arsenite, 5 μM cadmium chloride, 5 nM rotenone, and 300 nM tunicamycin. GW788388 (1 μM) was used as a negative control. **a**, **b** GFP signal of reporter cell line was measured over time course of 24 h by high-content imaging. The data represents the mean of three experiments ± SD. Statistical significance was analyzed by applying an ordinary one-way ANOVA followed by a Dunnett post hoc test. Significance codes indicate a *p*-adj: *** < 0.001, ** < 0.01, and * < 0.05. HMOX1 mRNA expression upon treatment is included for direct comparison of induction patterns. FI, fluorescence intensity; FC, fold change

In addition to transcriptomics, the HMOX1-GFP reporter function of the employed cell line (Snijders et al. 2021) was employed using high-content imaging (HCI) to analyze the temporal activation of the *HMOX1* gene (as representative gene of the Nrf2-oxidative stress response pathway) in response to all test compounds. Furthermore, we used cadmium chloride as a positive control as this induced *HMOX1* upregulation and Nrf2 oxidative stress response pathway activation in the PTL and the two human renal proximal tubular cell lines HK2 and RPTEC/TERT1 as previously described (Wilmes et al. 2011; Aschauer et al. 2015; Singh et al. 2021). Both sodium arsenite and cadmium induced GFP intensities in a time-dependent way, starting at 5 h after treatment and increasing over time (Fig. 3a, b). GFP intensity levels increased in a linear way with sodium arsenite treatment reaching the highest levels at 24 h, whereas intensity levels were lower in response to cadmium reaching a maximum at 13 h with levels remaining high until 24 h (Fig. 3a, b). Gene expression levels of *HMOX1* matched mostly the GFP signals, but could already be detected earlier (1 h of exposure) for both cadmium chloride and sodium arsenite. Interestingly, the maximum upregulation of HMOX1 mRNA was seen at 5 h after exposure for both compounds, with expression levels decreasing slightly at later time points for cadmium chloride, while expression levels remained high in response to sodium arsenite treatment. All other compounds did not induce the HMOX1-GFP reporter within 24 h of the experiment (Fig. 3a). A much lower mRNA expression of *HMOX1* was observed for tunicamycin and rotenone at later time points that were not captured by measuring GFP expression suggesting that the transcriptomics provide a more sensitive endpoint or that there is delay in GFP expression.

### Temporal gene expression alterations

**Fig. 4:**
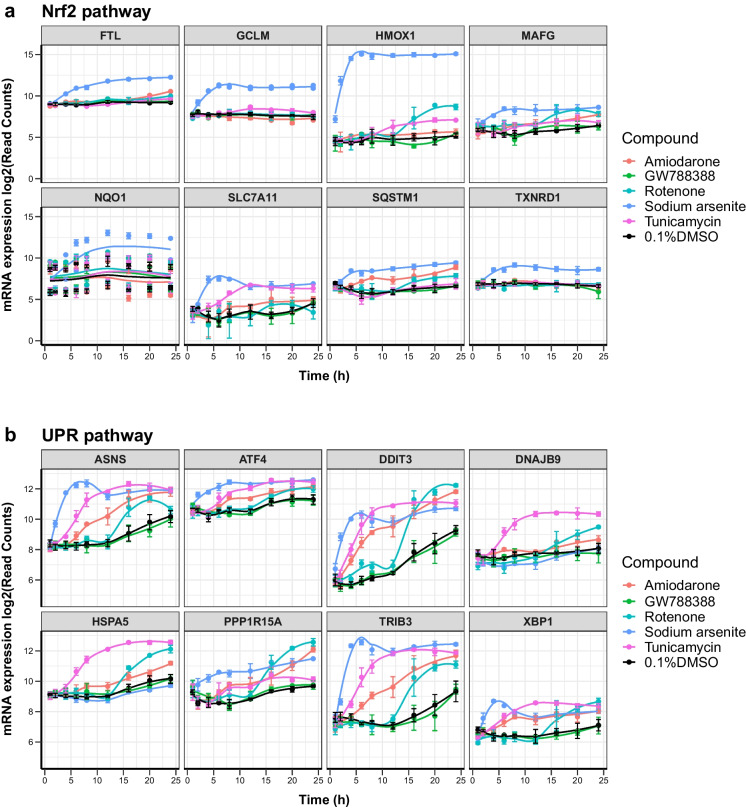

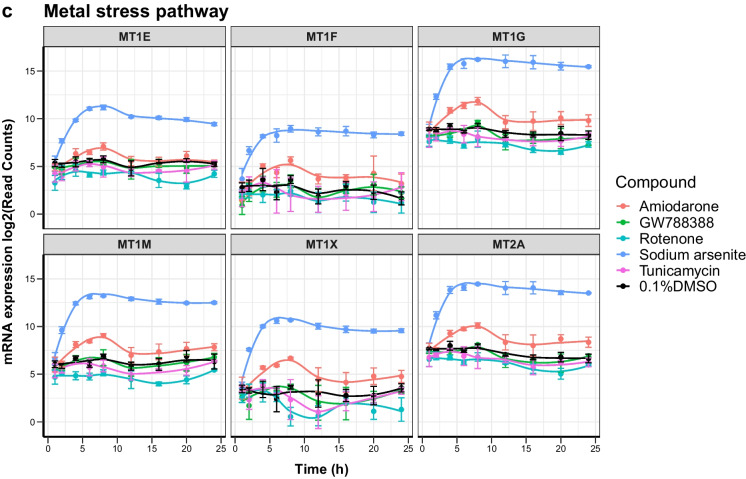
Temporal responses of mRNA expression of selected relevant genes. Time course representation of normalized mRNA read counts for representative genes per pathway. **a** Nrf2 oxidative stress response pathway, **b** unfolded protein response (UPR) pathway, and **c** metal stress response pathway. The data represents the mean of three experiments ± SD. Statistical significance was analyzed by applying an ordinary one-way ANOVA followed by a Dunnett post hoc test and are summarized in Table 1

**Fig.5:**
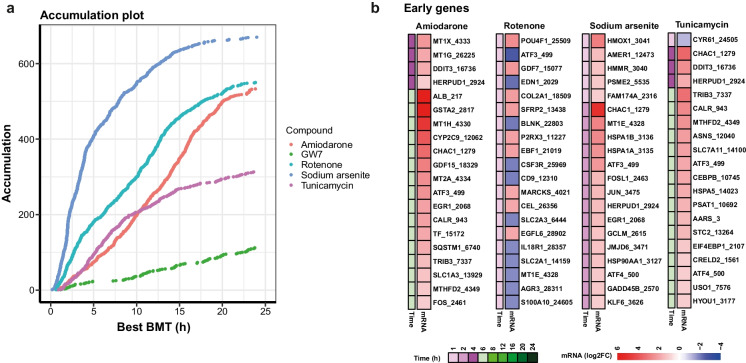
Determination of benchmark time (BMT) and early response genes. **a** Accumulation plots of best BMTs computed in BMDExpress2. 2713 out of 4911 probes were predicted to accumulate over time after applying the following cuts-off of significance: Best BMT between 6 min and 24 h (for better representation of realistic time points), BMTU/BMTL < 40, and fitPValue > 0.1. **b** Heat map representing earliest genes changed by compound, with significance cut-off of fold change > |2|, *p*-adj < 0.01

Temporal transcriptomics changes of selected representative genes, based on literature searches, for each of the three identified stress response pathways were also displayed in graphs showing read counts (Fig. 4) and significance values (Table 1). In addition, BMDExpress (Yang et al. 2007) was used to predict a benchmark time point (BMT) of the activation of the genes in the three pathways that were significantly changed in response to compound treatment. It should be noted that BMDExpress was developed to predict benchmark doses (BMDs) rather than BMTs and that the tool is generally employed to study the effect at one single time point and several different doses (or concentrations) of compounds. Here, rather having multiple concentrations, we entered multiple time points at a single concentration per compound. The predicted accumulation plot of all DEGs per compound is shown in Fig. 5a. Sodium arsenite had the earliest impact on PTL (approximately 400 genes within 5 h), followed by rotenone, amiodarone and tunicamycin. It should be noted though that accumulation over time is more complicated than an accumulation per concentration, as genes can be deregulated at an early time point and then come back to a basal expression level. Nevertheless, looking at the time course curves, one can observe a time-dependent increase in read counts of all representative genes within all the pathways. The responses either reach a plateau or decrease after the peak but maintaining a significant up/dowregulation; therefore, it is possible to assume a gene accumulation over time. BMDExpress predicted the earliest expression to be under 1 h for rotenone and sodium arsenite. By 5 h after treatment, a major accumulation of DEGs was already predicted for sodium arsenite, whereas for amiodarone and tunicamycin treatment, the 5 h time point seemed to be the earliest departure point with relatively few DEG accumulation. The earliest response gene per compound are shown in Fig. 5b. These included upregulation of genes within the metal stress pathway (*MT1X*, *MT1G*) and UPR pathway (*DDIT3*) in response to amiodarone, upregulation of genes within the Nrf2 pathway (*HMOX1*) and metal stress pathway (*MT1E*) in response to sodium arsenite that was similar to cadmium chloride reported earlier (Singh et al. 2021) and upregulation of genes within the UPR pathway (*DDIT3*, *TRIB3* and *ATF3*) in response to tunicamycin. Early responses to rotenone also included downregulation of several genes, including *ATF3* (UPR pathway) which was then upregulated at later time points. Table 2 shows the predicted BMT per stress response pathway in response to all tested compounds. Sodium arsenite treatment in PTL resulted in early BMT for activation of all three pathways and certain genes within each pathway were predicted to be activated > 1.5 h so that no clear pathways could be identified as being activated first. On the other hand, amiodarone treatment seemed to have an earlier BMT for the metal stress response (2–3 h) compared to the UPR stress response pathway (2–14 h). Interestingly, even though tunicamycin had an overall (all DEGs) late predicted BMT compared to rotenone, the specific impact on UPR stress response was predicted at a much earlier BMT (3–7 h) for tunicamycin compared to rotenone (12–17 h).

**Table 2:**
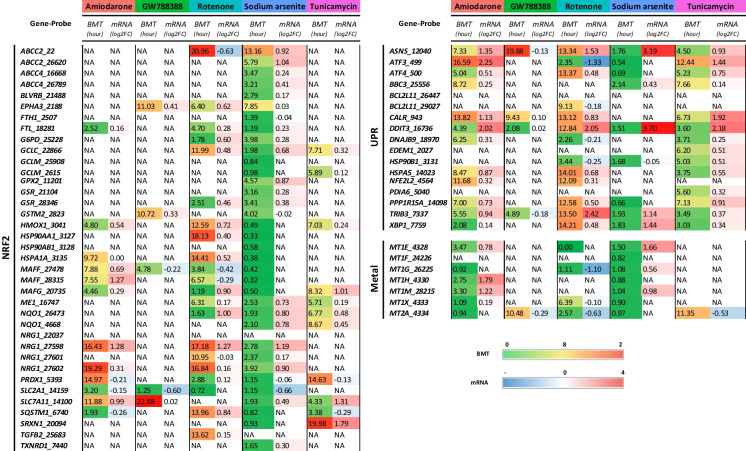
Predicted benchmark time (BMT) of selected genes per pathway and relative predicted log2FoldChange. BMT was calculated using BMDExpress software from the best fitted model; log2FoldChange was calculated in R using the weighted 5 − P logistic regression/progression model

### Alterations of gene expression levels in response to GW788388

**Fig. 6:**
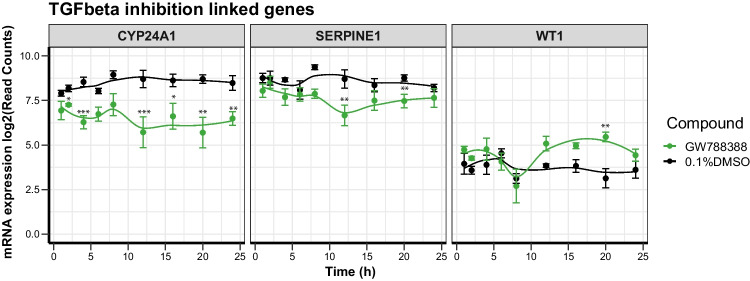
DEGs in response to GW788388 treatment (negative control) linked to TGFβ inhibition. PTL were differentiated in 96-well plate for 14 days and treated for up to 24 h with GW788388 (1 μM). Cells were lysed at different time points, and mRNA changes were analyzed by TempO-Seq transcriptomics. Total number of deregulated expressed genes (DEGs) were calculated by comparing with 0.1% DMSO-treated control cells. DEGs linked to TGFbeta inhibition are displayed over time course of 24 h. The data represents the mean of three experiments ± SD. Analysis of significance was performed by ordinary one-way ANOVA with Dunnett post hoc test. Significance codes indicate a *p*-adj: *** < 0.001, ** < 0.01, and * < 0.05

The TGFβ type I receptor kinase inhibitor GW788388 was used as a non-cytotoxic negative control compound in this study. It was used at 1 μM as this concentration is routinely used to extend stability and maintain differentiation status after sub-culture of PTL (passage 1) by maintaining tight junction proteins, including ZO3. Without GW788388, sub-cultured PTL rapidly lose their polarization, and ZO3 expression can be found in the nuclei rather than the tight junctions of the cells (Chandrasekaran et al. 2021). This compound is, however, not required for differentiated PTL that are not sub-cultured and hence also not applied to the PTL used in this study as these were used directly after differentiation (at passage 0). GW788388 did not induce any of the stress response pathways above and in fact had an overall low amount of DEGs (14 in total over all time points). Interestingly, out of these few DEGs, three DEGs could be connected to its mechanism of TGFβ inhibition: *CYP24A1* and *SERPINE1* showed decreased expression levels between 4 and 24 h and between 8 and 20 h after treatment, respectively, whereas WT1 showed increased expression levels after 20 h treatment (Fig. 6).

## Discussion

Transcriptomic studies in human cell systems have become an important tool to support the prediction of toxicity and the identification of potentially harmful substances. The possibility to screen for expression changes of thousands of genes simultaneously makes them attractive, and stress response pathways analysis tools help to decipher mechanisms of toxicity of test compounds. While the mainstay of toxicology is on concentration-dependent effects, temporal effects might also be useful to understand which responses are closer to the initial perturbation.

### Applicability of PTL in toxicity studies using transcriptomics

One of the aims of this study was to further evaluate the application of PTL cells for predicting toxicity using transcriptomics. Other iPSC-derived systems for different cell types had been successfully used in TempO-Seq transcriptomic studies, including iPSC-derived hepatocytes (ter Braak et al. 2021; Ghosh et al. 2021) and neurons (Dreser et al. 2020), and recently, we have reported a study employing iPSC-derived systems that were differentiated into kidney (podocyte-like and PTL cells), liver (hepatocyte-like cells), vasculature (endothelial-like cells), and brain (blood–brain-barrier-like cells, neuronal-like cells, and brain spheres) using TempO-Seq transcriptomics to predict mechanisms of toxicity in response to paraquat (Nunes et al., 2022). Paraquat is a well-described herbicide toxin that induces oxidative stress in the neurons, liver, and kidney (McCarthy et al. 2004; Onur et al. 2022) that was also picked up by iPSC-derived models in that study (Nunes et al. 2022). In addition to paraquat exposure, PTL cells had been previously reported to activate the Nrf2 oxidative stress response and metal stress response after treatment with the heavy metal cadmium chloride (Singh et al. 2021) at concentrations that had been previously reported to activate these pathways in the human proximal tubular cell lines HK2 (Wilmes et al. 2011) and RPTEC/TERT1 (Aschauer et al. 2015). In renal proximal tubular cells, the Nrf2-induced oxidative stress response is an important pathway that is often induced in response to many nephrotoxins, with *HMXO1* being one of the most upregulated genes (Wilmes et al. 2011, 2013; Aschauer et al. 2015). Other commonly induced stress response pathways were previously reported in RPTEC/TERT1 cells (Aschauer et al. 2015). Here, we treated PTL with additional compounds to study their effects on the activation of stress response pathways. The natural occurring metalloid sodium arsenite is often found in contaminated drinking water and has been linked to toxicity to every organ of the body; however, accumulation of sodium arsenite is often observed in the liver, kidney, and muscle (Chen and Costa 2021). Epidemiologic studies of sodium arsenite-contaminated drinking water showed a link for increased risk of developing chronic kidney disease (CKD) and end-stage renal disease (ESRD) and decreased estimated glomerular filtration rates (eGFR) and increased kidney injury marker 1 (KIM1) were observed (Farkhondeh et al. 2021). At the molecular level, sodium arsenite has been described to induce oxidative stress, alteration in DNA damage repair, and stimulation of p53 activation, mitochondria toxicity as well as changes to cytoskeleton and cell cycle (Medda et al. 2021). In this study, the response of sodium arsenite-treated PTL included the activation of metal stress response, oxidative stress response, and UPR response that was in line with previously reported mechanisms of sodium arsenite toxicity. Amiodarone is a widely used antiarrhythmic drug with toxicity observed in multiple organs, including the kidney (Morales et al. 2003), even though pulmonary toxicity has been described as its most severe adverse effect (Papiris et al. 2010), likely due to accumulation in alveolar type II lamellar bodies (Haller et al. 2018). In addition, animal and in vitro studies in the liver, kidney, and endothelial cells reported the involvement of oxidative stress (Sarma et al. 1997; Golli-Bennour et al. 2012). Furthermore, in iPSC-derived hepatocytes, the UPR pathway was one of the major impacted stress response pathways (Ghosh et al. 2021). PTL treated with amiodarone induced mainly pathways involved in inflammation, including interleukin 18- and NF-ΚB signaling and cytokine-cytokine receptor interaction (Fig. 2a). Several studies have reported a role of amiodarone in inflammation before, including a study using peripheral blood mononuclear cells (PBMCs) that showed downregulation of *TNFα*, *IL-6*, and *IL-1β* (Matsumori et al. 1997). In addition to an inflammatory response, several genes within the Nrf2 oxidative stress response and the unfolded protein response were upregulated significantly in PTL in response to amiodarone. Furthermore, we observed an activation of several genes within the metal stress response that had not been previously reported. This was an unexpected result, and we currently are working on building a hypothesis. The pesticide rotenone is a potent mitochondria complex I inhibitor that has been linked to the development of Parkinson’s disease, and it has been reported that rotenone may contribute to neurotoxicity by inducing oxidative stress as well as protein aggregation and degradation (Xiong et al. 2012). Recently, a study of one of our groups investigated the transcriptomics effects of rotenone in RPTEC/TERT1 cells and could show that the activation of the ATF4 branch of the UPR pathway was the major response observed after 24 h of exposure (Carta et al. under review). In the present study, UPR was also a main activated pathway in PTL after rotenone exposure, with *DDIT3* (aka CHOP) being the highest upregulated protein with the UPR pathway. Tunicamycin is a nucleoside antibiotic, originally isolated from *Streptomyces* species, that is well described for its inhibitory effect on UDP-GlcNAc and therefore often used as a model compound to induce ER stress and activation of the unfolded protein response pathways (UPR) (McMillan et al. 1994; Yan et al. 2018; Yamamoto and Ichikawa 2019). In PTL, tunicamycin also strongly induced all three branches of the UPR pathway and upregulations of *ATF6*, *ATF4*, and *XBP1* were observed. Finally, as expected, the negative control GW788388 did not induce any stress response pathway at 1 μM and had an overall very low impact on DEGs. GW788388 is a potent inhibitor of TGFβ type I receptor that had been discovered with the aim to development treatment for fibrosis (Gellibert et al. 2006) and has been shown to decrease EMT (epithelial-mesenchymal transition) and to reduce renal fibroses in mice (Petersen et al. 2008). While no transcriptomic studies have been reported for GW788388, interestingly, three of the DEGs changed in PTL after GW788388 treatment could be linked to its effect on EMT. Downregulation of *SERPINE1* has been shown to have an inhibitory effect on EMT, whereas high levels have been identified as a potential biomarker for EMT in gastric cancer (Yang et al. 2019; Xu et al. 2019). Wilms tumor 1 (*WT1*) has been discussed as a regulator of EMT during development and in disease (Miller-Hodges and Hohenstein 2012). More recently, knock down of *CYP24A1* has been described to have an inhibitory effect on EMT (Wang et al. 2020).

### Temporal effects and early response genes of common stress response pathways

Even though transcriptomics studies in the field of toxicology have increased significantly in recent years, no standardized methods, e.g., on time points for chemical exposure, exist. Studies that analyzed time points before 24 h of compound have been reported for human hepatocyte studies, including HepG2 cells treated with compounds that induce oxidative stress (0.5 to 24 h time points) (Deferme et al. 2013) as well for iPSC-derived PTL treated with cadmium chloride (1 h to 7 days) (Singh et al. 2021). In the study presented here, it was observed that temporal effects on activating stress response pathways were mainly compound-specific rather than pathway-specific. This was apparent for the activation of the UPR pathway that was impacted on early in response to tunicamycin (starting 3 h after treatment) and much later in response to rotenone (starting 12 h after treatment) (Table 2). Nevertheless, it was interesting that a sequential activation of genes and stress-response pathways was observed for some compounds, including amiodarone that increased genes within the metal stress response pathways first (majority of genes predicted to be deregulated below 4 h) and only later had an impact on the UPR pathway (only 2 genes deregulated early and majority predicted to be deregulated after 4 h) (Table 2). Hence, temporal expression profiles may help to identify the primary mechanism of toxicity and distinguish that from overlapping effects to other pathways. It has been previously reported that signatures between pathways, including Nrf2, UPR (ATF4 branch), and AhR, may overlap (Zgheib et al. 2018). This sequential activation was not observed in response to sodium arsenite that showed an early impact on all of the four stress-response pathways (Table 2). Activation of the Nrf2 oxidative stress response and metal stress response pathways by sodium arsenite, even though induced quite early, remained high throughout the time course of the experiment (Fig. 4a–c), whereas activation of the UPR pathway by tunicamycin and sodium arsenite (Fig. 4b) was the highest before the 24 h time point and decreased again during the time course of the experiment. It was noted that these effects differed between individual genes within one pathway and therefore highlight the need to judge this on a gene by gene basis. While transcriptomic study usually covers numerous genes per pathway, this may not be the case for biomarker panels based on fewer genes and should be taken into consideration. Furthermore, 24 h of exposure may not always be needed to detect activation of stress response pathways or genes within these pathways that may function as biomarkers. This was also in line with the results from the *HMOX1* reporter assay that was activated much earlier than 24 h after treatment of sodium arsenite and cadmium chloride (Fig. 3). We reported the early response genes in Fig. 5b. These may represent good candidates for the further investigation on development of fluorescent reporters or biomarker panels, even though other criteria for biomarkers or reporters have to be checked. Additionally, these early response genes may help decipher mechanistic information on initial events and distinguish these from overlapping downstream effects. Early response genes, include among others several *MT1* isotypes, *HMOX1*, *XBP1*, and *DDIT3*, and a compound specific list is given in Table 1.

## Conclusions

This study showed that iPSC-derived PTL could be successfully employed in TempO-Seq transcriptomic assays to capture the activation of commonly induced stress-response pathways, including Nrf2-oxidative stress, UPR, and metal stress pathway. Temporal analysis of gene expression levels and pathway enrichment showed that the effect of time was compound specific with strong responses often observed as early as 6–8 h after treatment. Additionally, for some compounds a sequential activation of different stress-response pathways could be detected, which will be helpful to build on the mechanistic unraveling of cell stress and adaptation.

## Supplementary Information

Below is the link to the electronic supplementary material.

Supplementary file1 (PDF 16138 KB)

Supplementary file2 (PDF 1245 KB)

## Data Availability

The datasets generated during and/or analyzed during the current study are available from the corresponding author on reasonable request.
