# Supplementary figures and images for: Capturing time-dependent activation of genes and stress-response pathways using transcriptomics in iPSC-derived renal proximal tubule cells
Source: Cell Biol Toxicol. 2022 Dec 31;39(4):1773–93. doi: 10.1007/s10565-022-09783-5 (PMC10425493; doi:10.1007/s10565-022-09783-5)

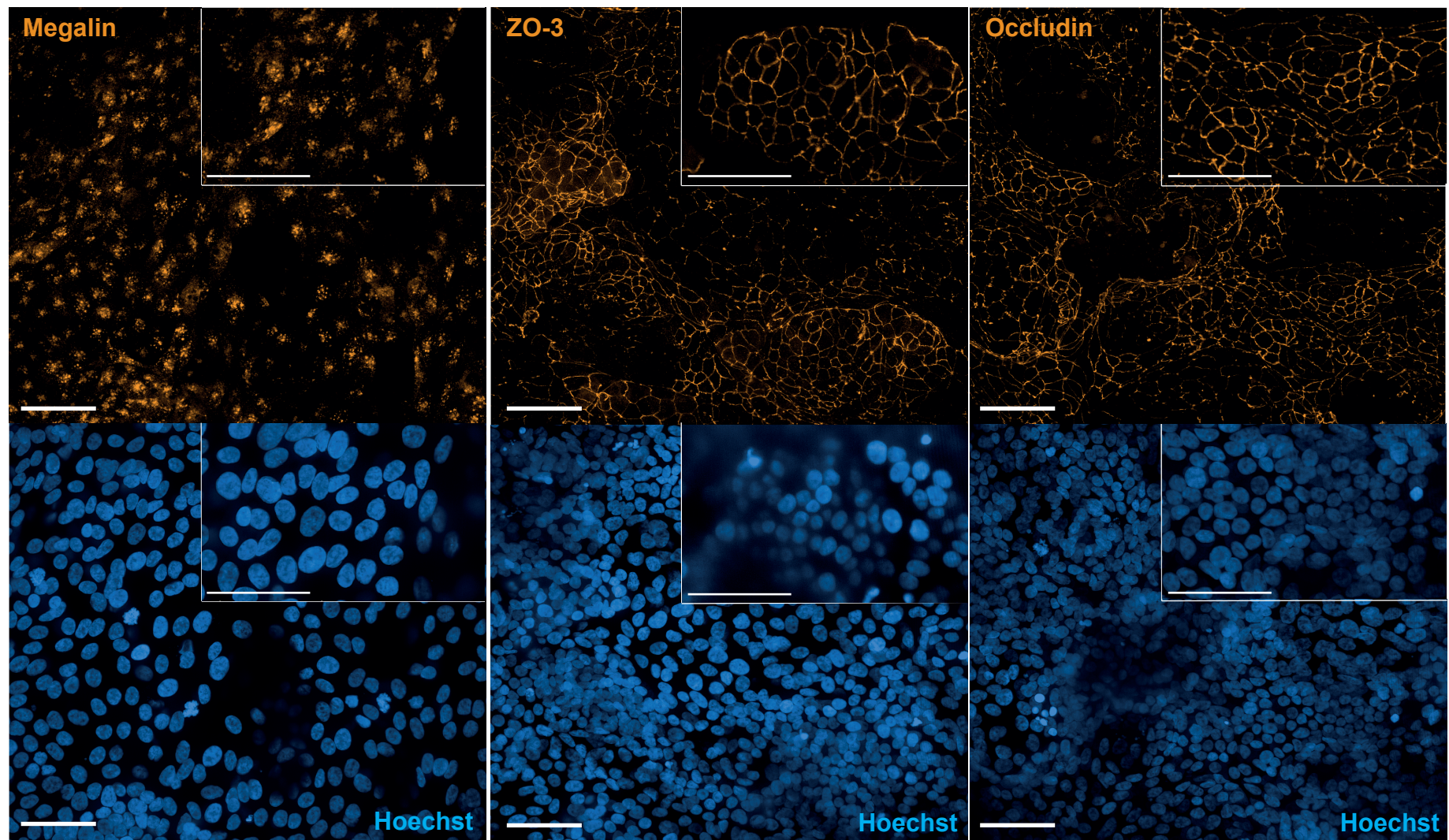

Supplement: Supplementary file 1 — Supplementary file1 (PDF 16138 KB) [file 10565_2022_9783_MOESM1_ESM.pdf]

Amiodarone

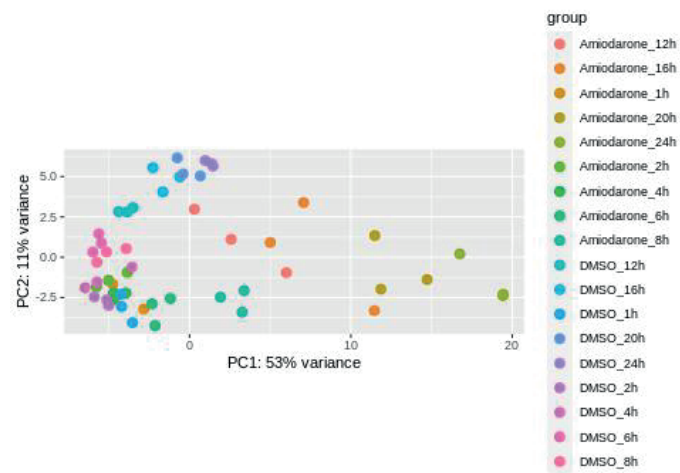

GW788388

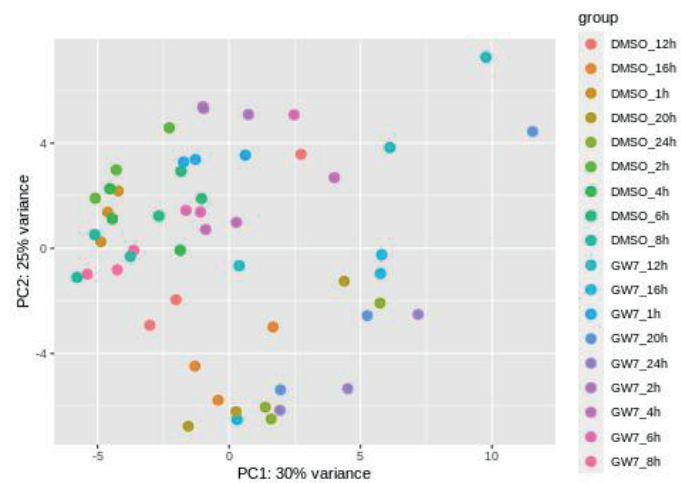

Rotenone

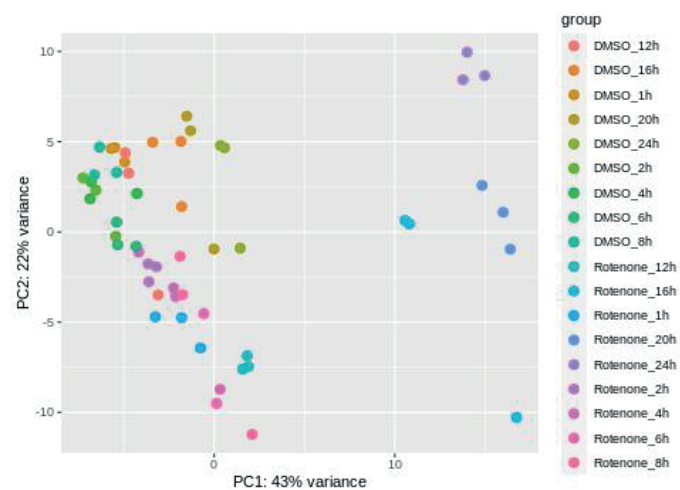

Sodium arsenite

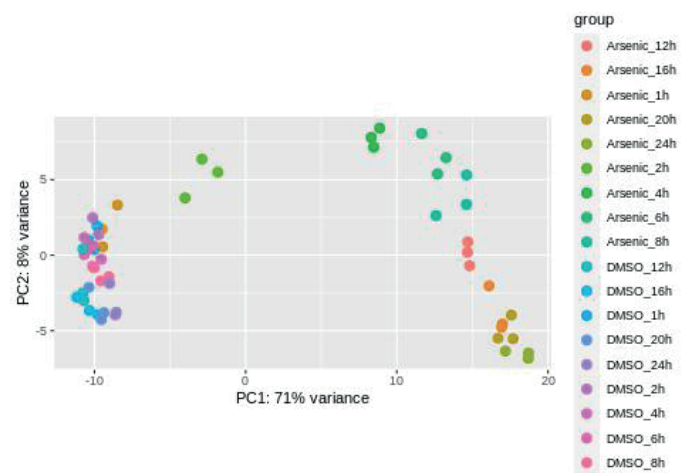

Tunicamycin

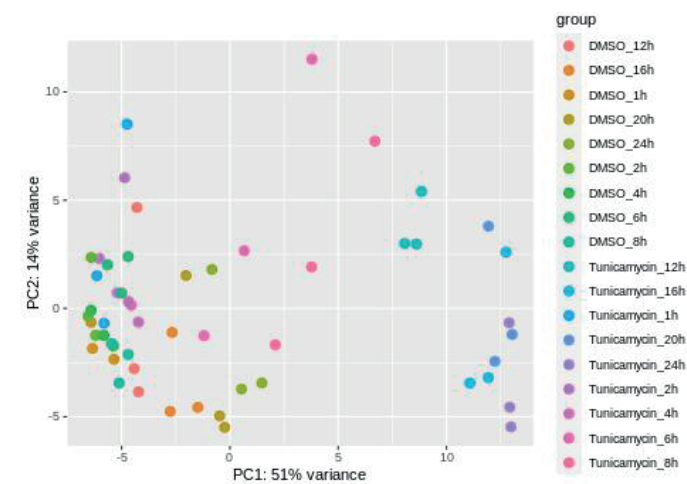

Supplement: Supplementary file 2 — Supplementary file2 (PDF 1245 KB) [file 10565_2022_9783_MOESM2_ESM.pdf]
